# Transthoracic Repair of Giant Paraesophageal and Hiatal Hernias: A Systematic Review and Meta-Analysis

**DOI:** 10.7759/cureus.74470

**Published:** 2024-11-26

**Authors:** Suhaib R Abdul, John T Fernando, Jonny R Varma, Syed Ifitikhar

**Affiliations:** 1 General Surgery, University Hospitals of Derby and Burton NHS Trust, Derby, GBR; 2 Orthopedic Surgery, North Bristol NHS Trust, Bristol, GBR; 3 Surgical Oncology, University Hospitals of Derby and Burton NHS Trust, Derby, GBR

**Keywords:** belsey mark iv fundoplication, hiatus hernia, minimally invasive laparoscopy, para-oesophageal hiatus hernia, robotic surgical procedures, thoracotomy procedure, vats video assisted thoracoscopic surgery

## Abstract

The utilization of transthoracic approaches for the repair of large hiatus hernias remains a topic of clinical debate. This study aims to evaluate the efficacy, safety, and recovery metrics for transthoracic hiatal hernia repair.

A literature search was conducted using the key terms "hiatus hernia," "thoracotomy," "thoracic approach," and "Belsey Mark IV." The databases searched included MEDLINE, EMBASE, and Web of Science, covering the period from 2000 to June 2024. Extracted data included patient demographics, study design characteristics, length of stay, complication rate, and mortality rate.

A total of five citations were selected, comprising a total of 560 patients, of which 164 were male (29.3%), with an overall mean age of 64.9 (pooled SD = 1.93) and a weighted mean follow-up length of 56.4 months (pooled SD = 39.1 months). The weighted overall mean length of stay was 14.5 days (pooled SD = 7.42). The overall rate of minor complications was 19% (95% confidence interval (CI) (6%, 31%)). The overall rate of major complications was 13% (95% CI (6%, 21%)). There were four reported mortalities in total and an overall leak rate of 1% (95% CI (0%, 2%)).

Transthoracic approaches have unique benefits and risks in the context of hiatal hernia surgery. Access via thoracotomy is associated with a higher incidence of complications. However, for large or emergent paraesophageal hernias, the transthoracic approach may represent a viable option in select patients.

## Introduction and background

Hiatal hernias, especially giant paraoesophageal hernias, pose a considerable clinical challenge due to their propensity to result in severe complications such as gastric mucosal necrosis, perforation, strangulation, erosive ulcers, and gastric volvulus [1]. The conventional surgical approach has predominantly involved laparoscopic techniques due to their minimally invasive nature and the associated shorter recovery period. With the advent of minimally invasive surgery (MIS), there has been an overall improvement in patient outcomes in terms of morbidity and mortality when compared to transthoracic and transabdominal open surgical procedures [2]. Nevertheless, in instances involving large or complex hernias (typically type III and type IV), the relative efficacy and safety of the laparoscopic approach, as compared to thoracotomy, continue to be matters of ongoing debate. However, since no randomized controlled trials have been performed to demonstrate which method (thoracoscopic vs laparoscopic) is superior, the choice of approach still depends largely on the preferences and skills of each surgeon, although the vast majority of surgeons currently employ the laparoscopic approach to treat hiatal hernias [3].

Transthoracic surgical approaches by way of thoracotomy have traditionally been utilized for the repair of large hiatal hernias, particularly in situations where the anatomical complexity or size of the hernia renders laparoscopic intervention challenging. Surgeons who advocate the preferential use of transthoracic repair argue that this method provides better visualization of the herniated structure and thus facilitates the dissection and resection of the sac [3]. Among the thoracic techniques, the Belsey Mark IV (BM-IV) procedure is notably one of the most recognized, and frequently employed when direct access to the esophagus and surrounding structures is necessary. Although the BM-IV is not widely performed, it is a favorable choice in patients with extreme obesity, large hiatal hernias, previous abdominal surgery, redo surgery, esophageal dysmotility, and extreme esophageal shortening [4]. However, the highly invasive nature of thoracotomy, along with the increased risk of complications, has generated divergent perspectives on its role within contemporary surgical practice.

Aims and objectives

This systematic review and meta-analysis aims to assess the outcomes of transthoracic repair of giant paraesophageal and hiatal hernias, with a particular emphasis on its efficacy, safety, and recovery parameters. By synthesizing data from observational studies and case series conducted over the past two decades, this review seeks to offer a comprehensive analysis of the advantages and risks associated with the thoracic approach, especially in the context of complex or emergent cases.

## Review

Materials and methods

Literature Search

A primary literature search was conducted using the key terms "hiatus hernia," "thoracotomy," "thoracic approach," and "Belsey Mark IV." A secondary literature search was also performed with similar key words, though this time considering minimally invasive transthoracic approaches, but data was not included in the meta-analysis. The new key terms were “video-assisted thoracoscopic surgery (VATS)” and "thoracoscopy." The databases searched included MEDLINE, EMBASE, and Web of Science, covering the period from 2000 to June 2024. The computer-based searches combined free-text and MeSH terms with keywords related to the population and intervention.

Inclusion and Exclusion Criteria

Abstract-only and poster presentations were excluded, and only studies in English were considered. The inclusion criteria required that all studies be observational and longitudinal in design, including prospective cohort, retrospective cohort, case-cohort, and nested-case cohort studies. Studies needed a population greater than 10 cases to be included in the review. Studies combining data for a transthoracic approach with another modality (laparoscopic or open abdominal) were excluded. Two reviewers independently searched databases and selected studies. Each article was evaluated against the inclusion criteria, and any disagreements about an article's eligibility were discussed. A consensus on inclusion was reached with the involvement of a third reviewer.

Quality Assessment

The methodological quality of the included studies was evaluated using the Newcastle-Ottawa (NOS) tool, a validated instrument for assessing the quality of observational studies [5]. Two independent reviewers conducted the quality assessments, and any discrepancies were resolved through discussion.

Data Extraction

Two independent reviewers performed the data extraction using a standardized and predesigned data collection form. They extracted available data on patient demographics (age, sex), study design characteristics (type of study design, follow-up duration), and relevant outcomes. If multiple publications existed for the same study, the report containing the most recent or comprehensive information was selected.

Data Synthesis

Data analysis and statistical figures were performed and extracted from Microsoft Excel (Microsoft Corporation, Redmond, Washington, United States) and the statistical package Jamovi ((Version 2.3) [Computer Software]. Retrieved from https://www.jamovi.org) [6]. Wan’s method was used to extrapolate means and standard deviations from medians and interquartile ranges [7]. Length of stay data underwent meta-analysis to a confidence interval (CI) of 95% and are presented as a forest plot. Incidence statistics underwent a meta-analysis of proportions, with the random effects model being used due to the relative heterogeneity in populations between studies. Mean age and continuous outcomes presented as raw data are represented as weighted mean difference (WMD). Heterogeneity was calculated for each meta-analysis using the I-squared (I^2^) statistic.

Results

**Figure 1:**
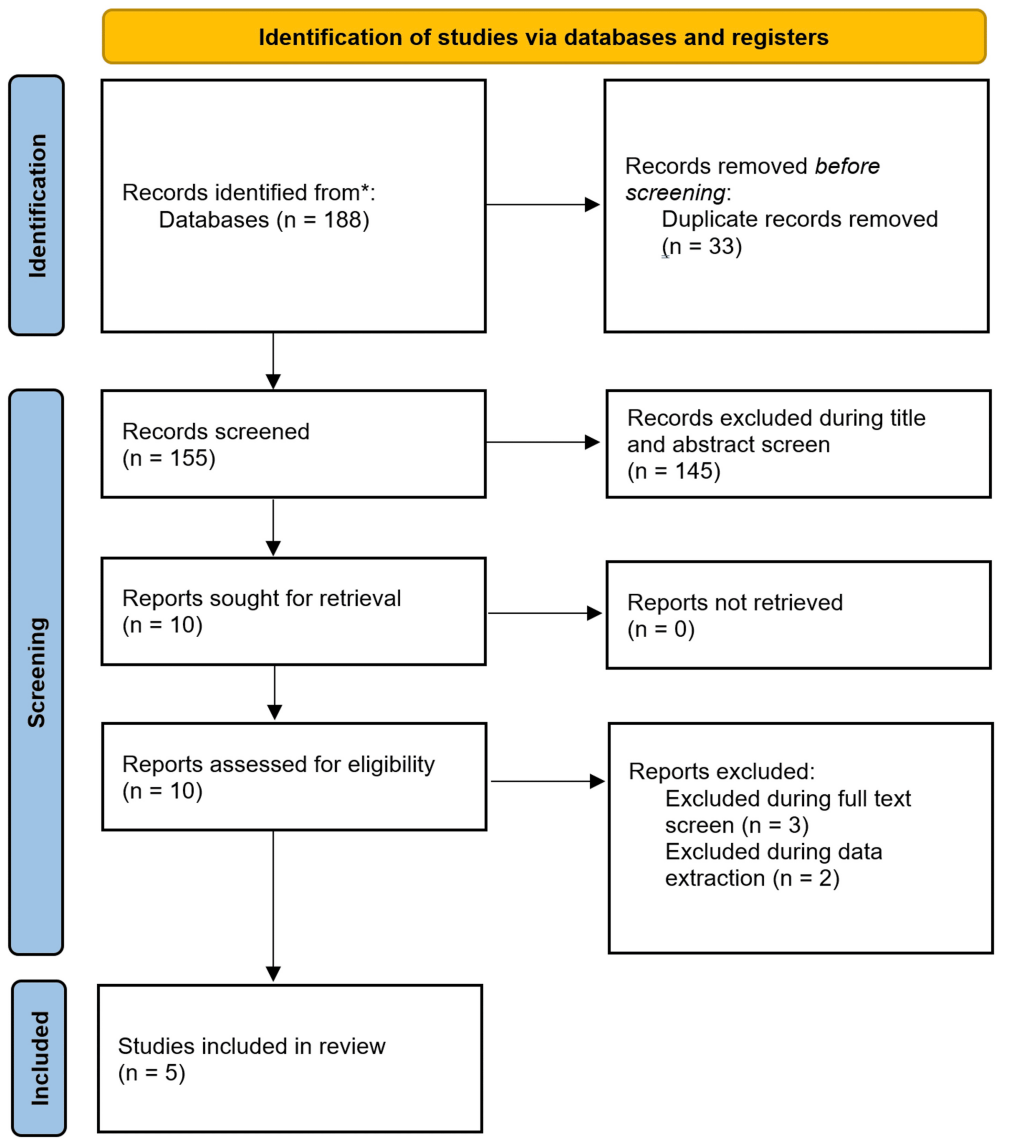
PRISMA diagram of the literature search * Databases used include MEDLINE, EMBASE, and Web of Science PRISMA: Preferred Reporting Items for Systematic Reviews and Meta-Analyses

Initial literature searches identified 188 references across all databases. We excluded 145 articles from the title and abstract search due to their lack of relevance to the core topic. Three studies were excluded during full text screening as they stated that they directly combined data on transthoracic approaches with other modalities. Two papers were excluded as it was not possible to directly extract transthoracic hiatal hernia repair data alone. A total of five studies were finally included for data analysis within the study (Figure 1) as per Preferred Reporting Items for Systematic Reviews and Meta-Analyses (PRISMA) guidance [8].

The overall median Newcastle-Ottawa score for the primary search was six. These scores equate to five articles being deemed good and the remainder fair in terms of quality by the Agency for Healthcare Research and Quality (AHRQ) standards [5,9]. The five citations included a variety of techniques of thoracotomy for repair, with only one study comparing results to conventional laparoscopic methods (Table 1). All studies were cross-sectional in nature.

**Table 1:** Citations used for analysis

Reference	Patients	Method	Comparison	Newcastle-Ottawa score
Ovaere et al. [10]	127	Belsey Mark IV (primary and redo)	None	7
Patel et al. [11]	240	Open primary thoracotomy	None	6
Laan et al. [12]	118	Primary Belsey Mark IV	Laparoscopic Nissen fundoplication	9
Rogers et al. [13]	60	Open primary thoracotomy	None	5
Markakis et al. [14]	15	Primary Belsey Mark IV	None	6

The overall analysis of all primary search publications demonstrated a total of 560 patients, of which 164 were male (29.3%) with an overall mean age of 64.9 (pooled SD = 1.93). Of the literature specifying the grade of paraesophageal hernia, the majority of procedures (98.4%) were performed on grade 3 or 4 hernias. The weighted mean follow-up length was 56.4 months (pooled SD = 39.1 months). The weighted overall mean length of stay was 14.5 days (pooled SD = 7.42). A breakdown of patient demographics is mentioned in Table 2.

**Table 2:** Demographic details of patients from each study * Data not available’ BMI: body mass index

Reference	Patients	Males (%)	Mean age (SD)	BMI	Reflux symptoms (%)	Epigastric or chest pain (%)	Dysphagia (%)	Dyspnoea (%)
Ovaere et al. [10]	127	24 (19%)	67 (8.3)	28.7	16 (12.6%)	58 (45.7%)	38 (29.9%)	28 (22%)
Patel et al. [11]	240	72 (30%)	63 (11.6)	30	165 (68%)	161 (67%)	87 (36%)	*
Laan et al. [12]	118	35 (30%)	69 (7.6)	30.5	5 (4.2%)	*	99 (83.9%)	5 (4.2%)
Rogers et al. [13]	60	22 (37%)	65 (10.4)	*	17 (28%)	22 (36.7%)	9 (15%)	14 (23.3%)
Markakis et al. [14]	15	11 (73&)	61 (11.8)	*	*	*	*	*

A meta-analysis of proportions was carried out for the rate of minor complications determined as a Clavien Dindo grade (Table 3) less than 3, with major complications being defined as a grade equal to or greater than 3.

**Table 3:** Clavien Dindo's classification of complications

Grade	Description
Grade I	Any deviation from the ordinary post-operative course without the requirement of any surgical or pharmacological intervention.
Grade II	Complications requiring pharmacological treatment.
Grade III	Complications requiring surgical intervention under local or general anesthesia.
Grade IV	Life-threatening complications requiring intensive care unit management.
Grade V	Complications leading to death.

**Figure 2:**
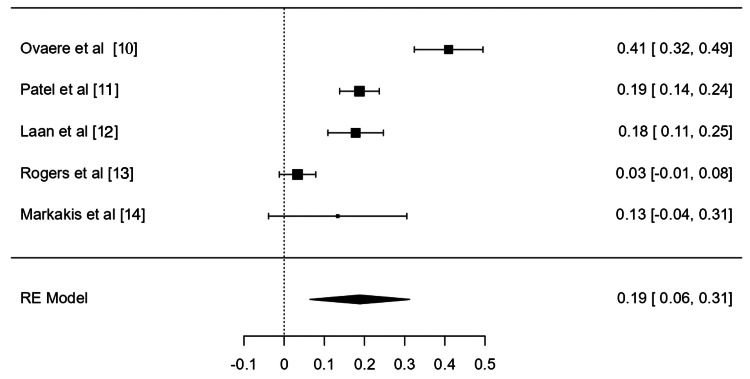
Meta-analysis of minor complication rates [10-14]

**Figure 3:**
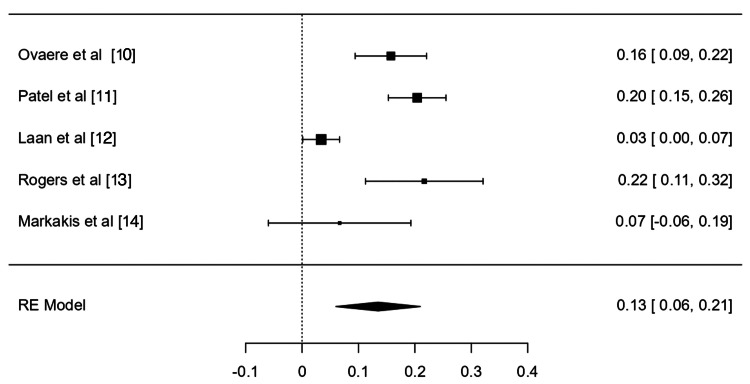
Meta-analysis of major complication rates [10-14]

The overall rate of minor complications (Figure 2) was 19% (95% confidence interval (CI) (6%, 31%)) with an I^2^ statistic of 94.01%. The overall rate of major complications (Figure 3) was 13% (95% CI (6%, 21%)) with an I^2^ statistic of 85.77%. The mortality rate was determined to be less than 1%, with only four total mortalities recorded in all studies. The leak rate was 1% (95% CI (0%, 2%)) with an I^2^ statistic of 0.0%.

The secondary search yielded four articles, of which three were contemporary and had available full texts. The overall median Newcastle-Ottowa score for the secondary search articles was seven and is therefore once again deemed good by the AHRQ standards [5,9]. Throascopic data was only presented in two citations and robotic transthoracic data in a single citation, thus making both these groups incompatible with meta-analysis.

Molena et al. and Derksen et al. both attempted to perform a combined thoracoscopic and laparoscopic procedure in order to repair large (grade IV or V) and often recurrent paraoesophageal hernias [15,16]. The two studies included a total of 21 patients, of whom eight were male with a combined mean age of 60.6 +/- 23.1. The pooled mean operative time was 349.1 +/- 99.4 minutes. The combined mean length of stay was 4.38 days +/-1.5. 

Reza et al. was the only citation describing a robotic transthoracic approach. The authors performed a robotic Belsey Mark IV procedure on a series of five patients with a reported mean operative time of 209 +/- 95 minutes and a mean length of stay of 4.2 +/- 2.8 days. The majority of patients (n = 3) in this case series had recurrent paraoesophageal hernias. 

Discussion

The findings of this systematic review offer valuable insights into the outcomes of thoracotomy for the repair of giant paraoesophageal and hiatal hernias, a subject of significant debate in recent years. The comprehensive analysis of 560 patients across multiple studies presents a nuanced understanding of the efficacy and associated risks of this surgical approach.

The results indicate that, although thoracotomy is associated with a relatively high complication rate, including minor (19%) and major (13%) complications, it remains a viable surgical option for patients presenting with large or complex hernias where laparoscopic techniques may be inadequate. Comparatively, the reported rates of minor and major complications of laparoscopic hiatal hernia procedures are <13% and <1%, respectively [17]. The higher incidence of complications compared to laparoscopic approaches is a crucial consideration; however, the overall mortality rate remains low (<1%), and the leak rate of 1% suggests that, in experienced hands, thoracotomy can serve as an effective intervention.

One of the key findings of this review is the weighted mean length of hospital stay, which was significantly longer at 14.5 days. This extended recovery period is likely reflective of the invasive nature of thoracotomy, which typically requires more intensive postoperative care and longer rehabilitation compared to minimally invasive techniques. The long-term follow-up data, with a weighted mean of 56.4 months, provides valuable information on the durability of the thoracotomy approach, with most cases involving high-grade hernias (grade 3 or 4).

The significant heterogeneity observed in the meta-analyses, particularly for minor and major complications (I^2^ of 94.01% and 85.77%, respectively), underscores the variability in patient outcomes and study designs, highlighting the need for careful patient selection and individualized treatment planning. This variability also suggests that while thoracotomy can be highly effective in certain scenarios, it may not be suitable for all patients, and the risks must be weighed against the potential benefits. One key advantage of the transthoracic approach is that the hernia sac may more easily be dissected and excised under vision [18].

Overall, this review supports the continued use of thoracotomy in specific clinical contexts, particularly for patients with complex or emergent paraesophageal hernias. However, the findings also emphasize the importance of ongoing research to further refine the indications for thoracotomy and to explore ways to minimize its associated risks. Thoracotomy provides excellent exposure of the esophagus and hernia sac, facilitating dissection of sufficient esophageal length and the possible creation of a gastroplasty to lengthen the esophagus if needed to ensure a tension-free repair [19]. 

One of the primary benefits of transthoracic paraoesophageal repair is the low long-term recurrence rate, demonstrated by several single-center series. At two to eight years of follow-up, patients report “good” or “excellent” results in 83-93% of cases. Radiologic recurrence rates are under 10%, and the reoperation rates are under 3% [20].

Future studies should focus on direct comparisons between thoracotomy and laparoscopic approaches in well-defined patient populations, as well as on the development of enhanced recovery protocols to reduce the length of hospital stay and improve postoperative outcomes.

One of the studies retrospectively reviewed five cases performed with robotic assistance, citing enhanced dexterity and precision in the transthoracic approach [21]. The patients, predominantly male with an average age of 64.4 years, included those with prior hiatal hernia repairs and one who underwent concurrent pulmonary surgery. The average operative time was approximately 209 minutes, with a mean postoperative stay of 4.2 days. Complications were minimal but included bleeding and an air leak post-lobectomy. The findings suggest that the robotic approach facilitates a safe and effective minimally invasive Belsey Mark IV repair, even in anatomically complex cases.

## Conclusions

Thoracotomy remains an essential option in the surgical repertoire for managing giant paraoesophageal and hiatal hernias, but its use should be judiciously evaluated, with particular attention to patient-specific factors and the risk of complications. The decision to utilize a thoracic approach must be grounded in a comprehensive assessment of the individual patient's condition, the surgeon's expertise, and the range of available surgical alternatives, ensuring that the selected method achieves the optimal balance between efficacy and safety. In recent years, thoracoscopic and robotic transthoracic approaches have gained attention as promising alternatives to traditional thoracotomy for the repair of giant paraoesophageal and hiatal hernias. These minimally invasive techniques leverage advanced technology to enhance surgical precision and control, allowing for a more targeted intervention with less disruption to surrounding tissues. When performed by skilled and experienced surgeons, these approaches have demonstrated the potential to significantly reduce postoperative recovery times, leading to shorter hospital stays and quicker returns to normal activities for patients. Thoracoscopic and robotic techniques represent an important area of ongoing research and clinical application, with the potential to refine and expand the treatment options available for patients with challenging paraoesophageal hernias. One of the main limitations of this study is a lack of comparative studies considering both laparoscopic and transthoracic approaches. More data is required to more directly compare transabdominal approaches to transthoracic approaches to further determine the most ideal utilization of transthoracic repairs. As robotic approaches become more feasible, there is a hope that further data may be published to compare various minimally invasive techniques.
